# Abnormal descent of the testis and its complications: A multimodality imaging review

**DOI:** 10.4102/sajr.v22i1.1374

**Published:** 2018-09-27

**Authors:** Pankaj Nepal, Devendra Kumar, Vijayanadh Ojili

**Affiliations:** 1 St. Vincent’s Medical Center, Connecticut, United States; 2 Hamad Medical Corporation, Doha, Qatar; 3 Department of Radiology, University of Texas Health, San Antonio, United States

## Abstract

Cryptorchidism refers to an absence of the testis in the scrotal sac. Testicular descent occurs in two stages: transabdominal and gubernacular. The descent of the testis can be arrested in its usual path of descent (true undescended testis) or can migrate from the usual path of descent (ectopic testis). Localising the missing testis is important for surgical planning, as well as for identification of complications that are more common with cryptorchidism. Ultrasound is the initial imaging modality to visualise, as well as localise the testis in cryptorchidism. However, ultrasound imaging is limited in visualising testes that are not superficial in location. This article highlights various examples of abnormal descent of the testis in usual as well as unusual locations and complications of undescended testes. Further evaluation with computed tomography scan or magnetic resonance imaging is needed in indeterminate cases and for identification of complications. We have highlighted the role of specific modalities with imaging findings in this pictorial review for the appropriate selection of each modality in clinical practice.

## Introduction

An absence of the testis in the scrotal sac is defined as cryptorchidism. The true undescended testis has arrested migration along its usual path of descent, or it is termed ectopic testis when it migrates from its usual path of descent to lie in an unusual location. An atrophic or congenital absence of the testis may simulate a similar situation. In infants, correct localisation of the testis is essential for surgical management, because the approach may vary with the location. In adults, however, it is still important to localise the testes and identify the complications.

Undescended testes are seen in approximately 1% – 6% of newborn males. The incidence is even higher in preterm infants, reported at 30%.^1^ Most undescended testes migrate into the lower scrotum within the first 3 months of life, probably as a consequence of a postnatal surge of testosterone. Only in less than 1% of the cases does the testis remain persistently undescended by the age of 1 year.^1^ Cryptorchidism occurs four times more commonly unilaterally than bilaterally.

Around 70% of undescended testes are palpable on clinical examination. Clinically, it can sometimes be difficult to distinguish an undescended testis from a retractile testis, which is excessively mobile.^2^

## Discussion

In 80% of patients with cryptorchidism, the testis is manually palpable in the inguinal canal.^3^ The normal course of testicular descent is retroperitoneally from the inferior pole of the kidney to the scrotum.^4^ The testis shares its embryological development with the kidney, and thus, it initially develops in the upper abdomen and migrates towards the inguinal canal through the deep inguinal ring after 21 weeks of gestation. The migration is almost complete at 30 weeks of gestation. Various factors influence the descent: hormones, including gonadotropins and testosterone, as well as active migration of the gubernaculum, determine its final position. The gubernaculum is the ligament that connects the developing testis to the scrotum and is responsible for inguinoscrotal descent. Under the influence of hormones (e.g. testosterone), the gubernaculum contracts and the testis descends into the scrotum. The gubernacular phase is more prone to error, whereas the transabdominal phase of arrest accounts for 5% – 10% of cases. On imaging, cryptorchidism can be broadly divided into two groups: arrested descent or ectopic testes.

### Arrested descent

Descent of the testes is along the normal path, but incomplete. The testis may be located in the inguinal canal (80%), near the pubic tubercle or, uncommonly, in the abdomen. The testis is often small and abnormal with a short spermatic cord.

### Ectopic descent

**FIGURE 1:**
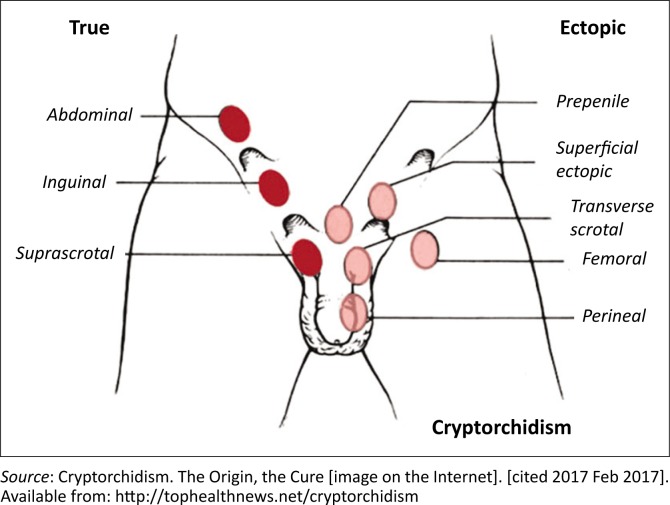
The most frequent locations of true and ectopic undescended testis.

Descent of the testes is away from the normal path. The testis is most often found in the superficial inguinal pouch. Other uncommon locations are perineal, abdominal wall, pelvic, crural, penile and femoral. The testis and spermatic cord are usually normal. Most frequent locations to aid a directed search for the testicles are shown in Figure 1.^6^

### Imaging modalities

**FIGURE 2:**
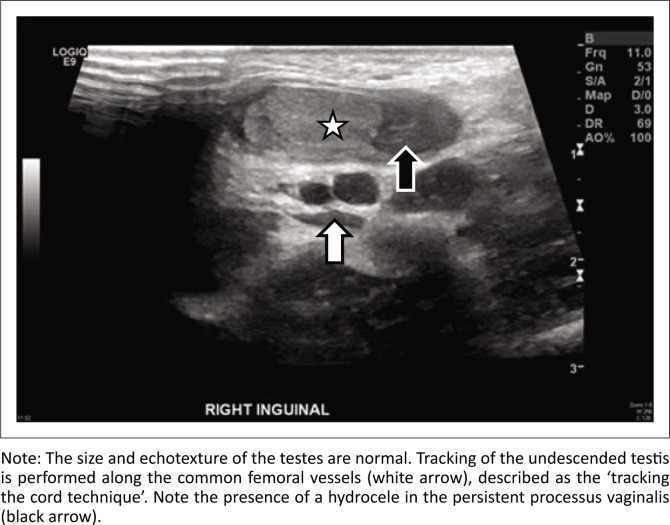
Greyscale ultrasound in a 6-month-old infant showing an undescended testis in the right inguinal canal (star).

The purposes of imaging are to correctly localise the testis and assess viability and size (assess for atrophy). The aim is also to detect cases of vanished testis or agenesis. Ultrasound is an initial imaging modality to localise the testes, as well as to assess vascularity on colour Doppler imaging. If the testis is not identified in the scrotum on ultrasound, then the ‘tracking the cord’ technique is useful.^5^ Starting below the inguinal crease, the common femoral artery and vein can be traced with the ultrasound probe in the transverse axis (Figure 2). The spermatic cord is identified in the inguinal canal, seen as an oval echogenic structure, anteromedial to the common femoral vessels. The common iliac and internal iliac vessels can then be traced cranially up to the bifurcation of the aorta to look for an abdominal testis. A superficial scan of the iliac fossa and pelvis is also performed to search for the testes.

**FIGURE 3:**
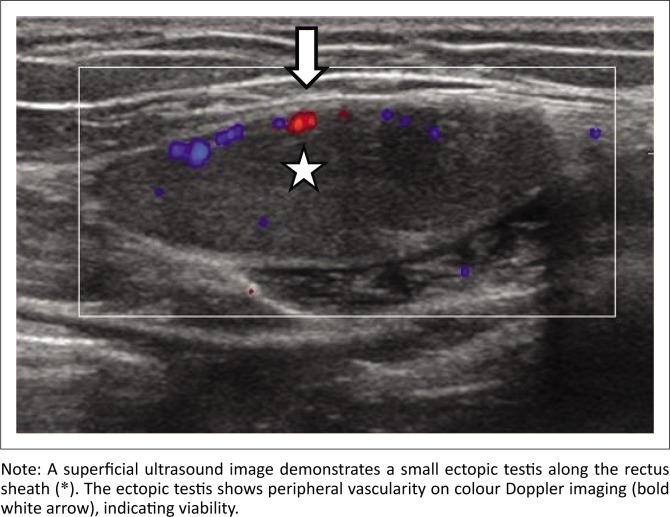
Sagittal ultrasound image of the left upper abdomen with a linear probe in a 2-year-old male child with left cryptorchidism.

Ultrasound is particularly useful in visualising superficially localised testes in the inguinal canal, inguinal pouch and subcutaneous location. Ultrasound has approximately 40%–50% sensitivity, 70%–80% specificity and around 88% accuracy for localisation of an undescended testis.^7^ It has superior resolution in demonstrating the superficial location of testes, such as along the rectus sheath, inguinal canal or perineum. Colour Doppler imaging is excellent in demonstrating vascularity within the testes (Figure 3). The limited accuracy of ultrasound is because of difficulty in visualising intra-abdominal, pelvic, retroperitoneal or ectopic testes (20%).^8^ Another limitation of ultrasound is failure to differentiate an atrophic testis from a lymph node or the gubernaculum.

Cross-sectional imaging modalities like computed tomography (CT) scans and magnetic resonance imaging (MRI) are used in indeterminate cases on ultrasonography, as well as in the assessment of complications. The testes appear hypodense on CT, and it may be difficult to differentiate them from lymph nodes or small cystic structures. CT scan involves significant radiation and raises concerns regarding the radiation dose as the majority of imaging for cryptorchidism occurs in the paediatric age group. CT scan is, however, superior in evaluating and staging known cases of testicular malignancy.

**FIGURE 4:**
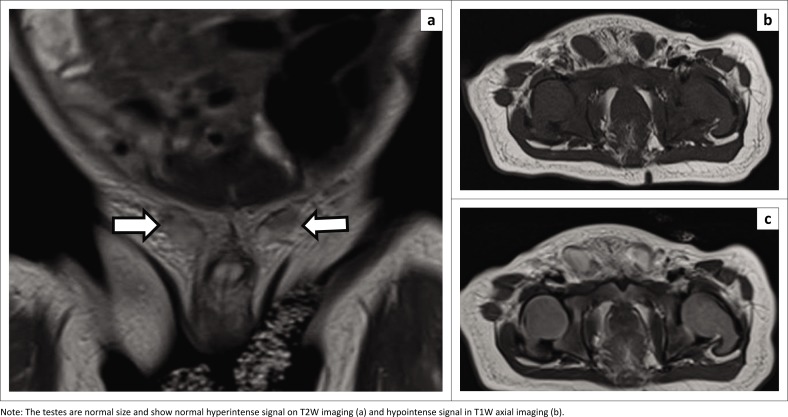
(a) Coronal T2-weighted (T2W), (b) T2W axial and (c) T1-weighted (T1W) axial magnetic resonance images in a 12-month-old male with bilateral undescended testes, demonstrating ectopic testes at the superficial inguinal ring (white arrows).

**FIGURE 5:**
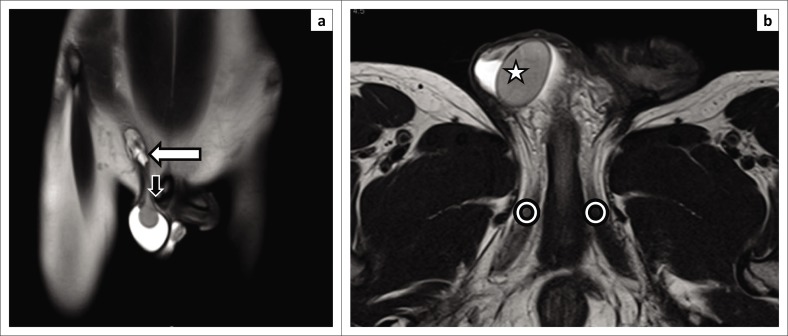
(a) Coronal and (b) axial T2-weighted (T2W) magnetic resonance images in 9-month-old male child showing true undescended right testis (star) at the level of the root of penis, near the pubic tubercle (black arrow). The crura of the penis are seen at this level (circles). The inguinal canal (white arrow) is well visualised on coronal section.

**FIGURE 6:**
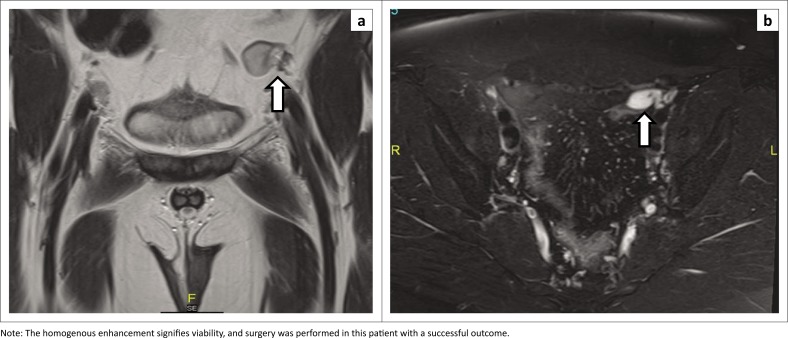
Use of contrast-enhanced imaging in a 7-month-old male with an undescended left testis: (a) Coronal T2W image shows a true undescended testis in the left iliac fossa (bold white arrow). (b) Axial post-contrast subtraction image confirms a homogenously enhancing undescended left testis (bold arrow).

Magnetic resonance imaging has a higher sensitivity of approximately 90% and specificity of almost 100% in localising the testes compared to ultrasound.^9^ Plain and contrast-enhanced Coronal T1-weighted (T1W) MRI can differentiate the gubernaculum, testes and spermatic cord. The spermatic cord can be followed to locate the undescended testes. The ectopic, pelvic or retroperitoneal location of the testes can be easily identified (Figure 4). The testis normally appears hyperintense on T2-weighted (T2W) MRI. Recently, diffusion-weighted MRI has been used to show a markedly hyperintense signal within testes, which helps to differentiate it from lymph nodes and surrounding structures.^10^ Contrast-enhanced MRI is also better for visualisation, demonstrating testicular enhancement, which is also indicative of viability (Figures 4, 5, and 6).

If until the age of 6 months (corrected for gestational age) a testis has not descended, surgery should be performed within the subsequent year, preferably by the age of 18 months. Histological examination proves that undescended testes suffer a progressive loss of germ cells as well as Leydig cells.^11^ Prepubertal orchidopexy addresses the issue of infertility, and there has been worldwide evidence that it may decrease the risk of testicular cancer in children with cryptorchidism.^12^

### Imaging algorithm

Seventy per cent of undescended testes are palpable manually, and in the remaining 30% of non-palpable undescended testis, the current algorithm is to perform abdominal-scrotal ultrasound first. Ultrasound is sensitive in visualising testes in the inguinoscrotal region or localised to the superficial abdomen wall and perineum. If the ultrasound findings are non-diagnostic, the next imaging modality is MRI. CT is non-invasive but is unreliable in identifying the testes and carries the risk of radiation. As per international guidelines from the American Association of Urology (2014), imaging in cryptorchidism is currently advocated as an adjunct. According to their guidelines, diagnostic laparoscopy or open exploration must be performed on all non-palpable cryptorchid patients.^13^ This should, however, take into account the local expertise and practice available. Viable and normal size undescended testes are treated with orchidopexy, depending upon expertise, with laparoscopy or an open surgical approach. A small and atrophic intra-abdominal testis can be treated with orchiectomy.

**FIGURE 7:**
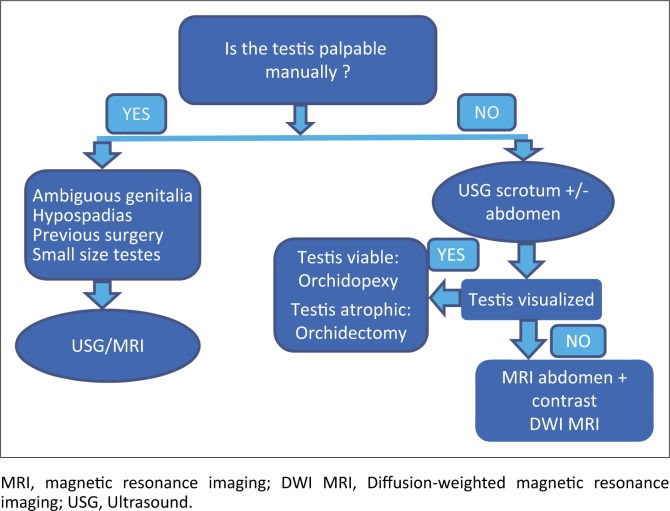
Imaging algorithm in undescended testis.

Preoperative localisation of the testis definitely aids in surgical planning and approach.^14^ It may reduce the extent of exploration and time for anaesthesia. Accurate pre-surgical localisation of the testis can provide the surgeon with the anatomic knowledge to tailor the operative approach. MRI has almost 100% specificity in identifying and localising the testes. Laparoscopy and surgery are difficult in children who have had previous inguinal or scrotal surgery because of scarring, increased risk of injury and reduced mobility of the spermatic cord. Cryptorchidism associated with ambiguous genitalia or hypospadias also need mandatory imaging to evaluate for the presence of Mullerian structures. The authors feel that this topic has not received much attention in the literature recently. This then begs the question that in the absence of visualisation of testes with MRI, is it logical to undergo surgery? Further research into the role of imaging in undescended testis is necessary before concluding imaging just as an adjunct. A simple imaging algorithm for cryptorchidism has been designed by the authors and illustrated in Figure 7.

### Complications

**FIGURE 8:**
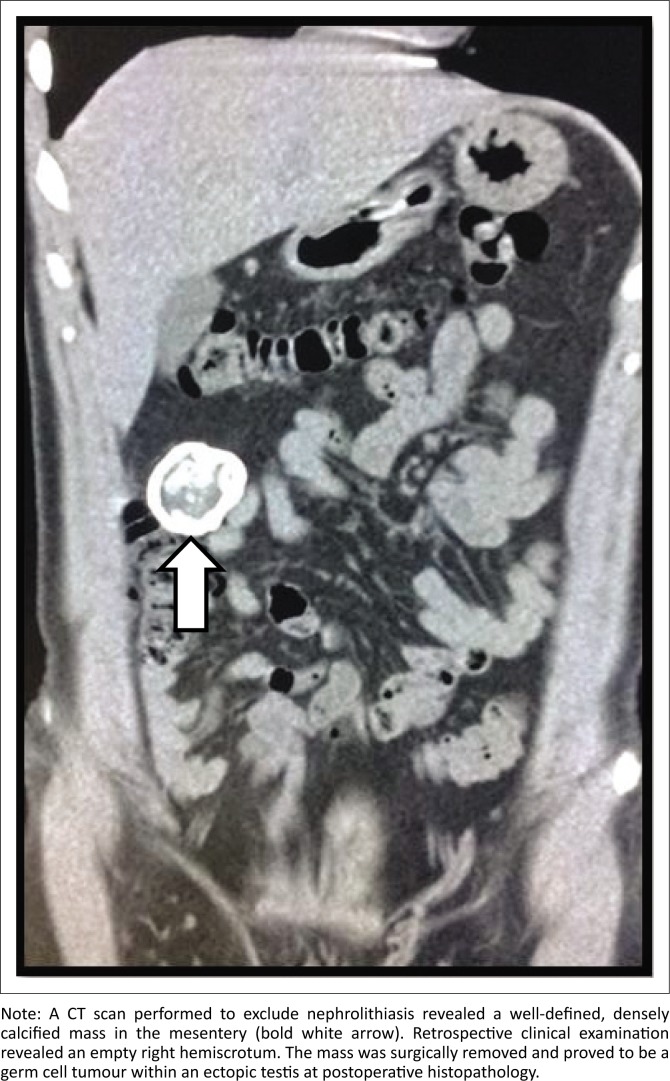
Coronal non-contrast computed tomography abdomen of a 35-year-old male who presented to the emergency department with right flank pain.

**FIGURE 9:**
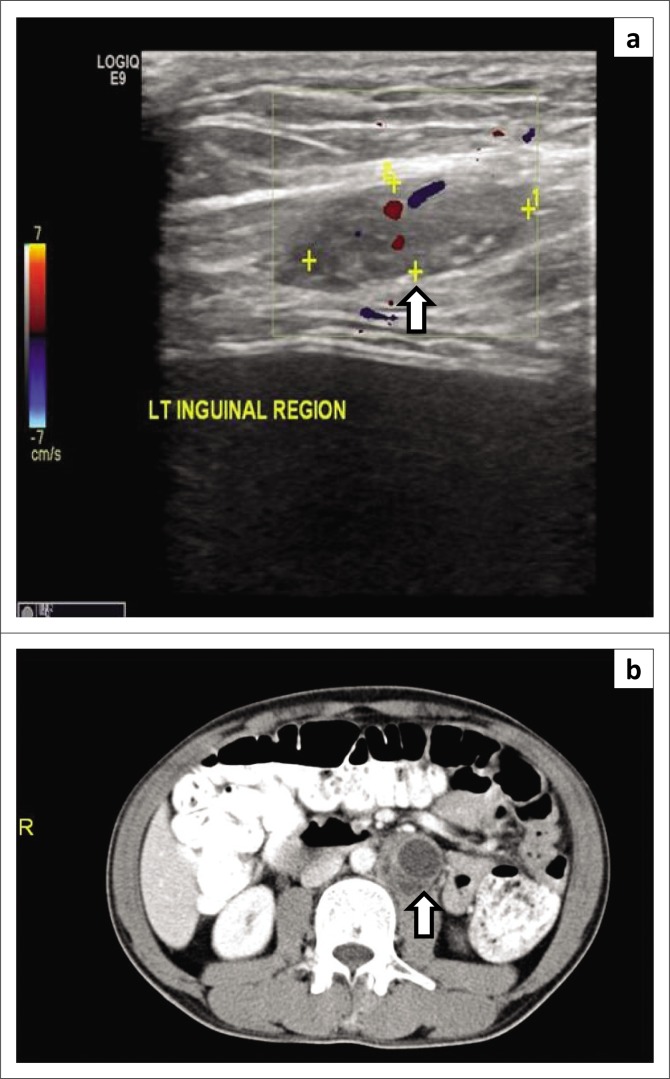
A 30-year-old male patient with a left-sided undescended testis: (a) Ultrasound imaging in the left inguinal location showed an atrophic and echogenic testis (bold white arrowhead). Postoperative findings were consistent with testicular non-seminomatous germ cell tumour and epididymis disjunction. (b) Contrast computed tomography for systemic work-up revealed necrotic heterogeneous lymph nodal metastasis (bold white arrow) in the left para-aortic location, which regressed after chemotherapy.

Complications of undescended testes include an increased incidence of infertility, trauma, malignancy, torsion, other associated anomalies, as well as inguinal hernia. Although there has been dispute regarding orchiopexy reducing the risk of testicular cancer, it certainly increases the detection of malignancy through testicular self-examination.^15^ The incidence of testicular malignancy among men with an undescended testicle is approximately 1 in 1000–2500, which is certainly higher than the normal population. Testicular malignancy has been reported in 10% – 15% of patients with undescended testes^16^ (Figures 8 and 9).

**FIGURE 10:**
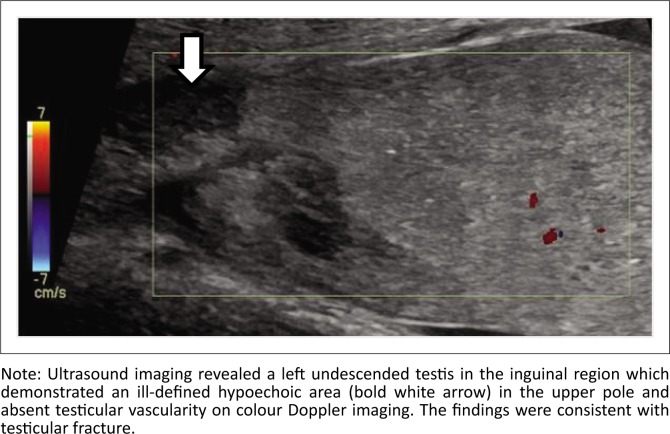
A 15-year-old boy with trauma to the inguinal region presented to the emergency department.

**FIGURE 11:**
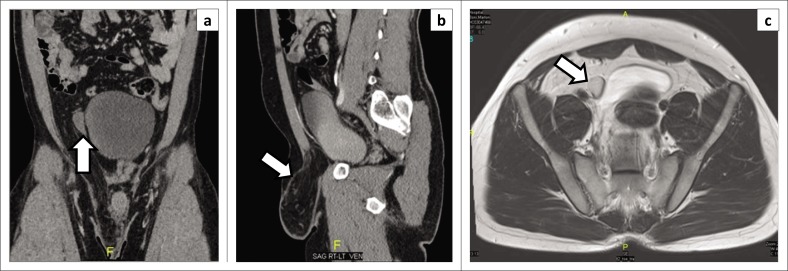
A 30-year-old male presented to the emergency department with pain in the right inguinal region with a large inguinoscrotal hernia on clinical examination: (a) Coronal and (b) sagittal images of a computed tomography abdomen demonstrated an undescended testis (arrow on image a) along its path in the right lower abdomen, located lateral to the surface of urinary bladder (U). There was an associated large right omental hernia (arrow on image b). (c) The undescended testis is hyperintense (arrow on image c) on axial T2-weighted magnetic resonance images.

If a testicle is located in the inguinal location, it might be prone to trauma or pressure against the pubic bone because of its superficial location (Figure 10). The incidence of testicular torsion is thought to be higher in undescended testes than in normal scrotal testes.^11^ Torsion of an undescended testis can be attributed to the development of a testicular tumour, increasing the weight and distorting the normal anatomy of the organ. Torsion of an intra-abdominal testicle may present as an acute abdomen. Inguinal hernias may be associated with the cryptorchidism^17^ (Figure 11).

## Conclusion

Clinicians and radiologists should be aware of the chronology of normal testicular descent, the common and uncommon locations of cryptorchidism, the specific imaging approach, as well as the complications associated with undescended testes.
